# Design of Injectable Bioartificial Hydrogels by Green Chemistry for Mini-Invasive Applications in the Biomedical or Aesthetic Medicine Fields

**DOI:** 10.3390/gels9010059

**Published:** 2023-01-11

**Authors:** Rossella Laurano, Monica Boffito, Claudio Cassino, Francesco Liberti, Gianluca Ciardelli, Valeria Chiono

**Affiliations:** 1 Department of Mechanical and Aerospace Engineering, Politecnico di Torino, 10129 Torino, Italy; 2 Institute for Chemical-Physical Processes, National Research Council (CNR-IPCF), 56124 Pisa, Italy; 3 Department of Science and Technological Innovation, Università del Piemonte Orientale, 15121 Alessandria, Italy

**Keywords:** bioartificial hydrogels, injectable systems, green functionalization procedures, in situ cross-linking, mini-invasive applications

## Abstract

Bioartificial hydrogels are hydrophilic systems extensively studied for regenerative medicine due to the synergic combination of features of synthetic and natural polymers. Injectability is another crucial property for hydrogel mini-invasive administration. This work aimed at engineering injectable bioartificial in situ cross-linkable hydrogels by implementing green and eco-friendly approaches. Specifically, the versatile poly(ether urethane) (PEU) chemistry was exploited for the development of an amphiphilic PEU, while hyaluronic acid was selected as natural component. Both polymers were functionalized to expose thiol and catechol groups through green water-based carbodiimide-mediated grafting reactions. Functionalization was optimized to maximize grafting yield while preserving group functionality. Then, polymer miscibility was studied at the macro-, micro-, and nano-scale, suggesting the formation of hydrogen bonds among polymeric chains. All hydrogels could be injected through G21 and G18 needles in a wide temperature range (4–25 °C) and underwent sol-to-gel transition at 37 °C. The addition of an oxidizing agent to polymer solutions did not improve the gelation kinetics, while it negatively affected hydrogel stability in an aqueous environment, suggesting the occurrence of oxidation-triggered polymer degradation. In the future, the bioartificial hydrogels developed herein could find application in the biomedical and aesthetic medicine fields as injectable formulations for therapeutic agent delivery.

## 1. Introduction

Hydrogels are three dimensional (3D) highly hydrophilic networks able to resemble the composition of the native extracellular matrix due to their high water content [1,2]. Such a feature makes these systems extensively exploited in the biomedical field as versatile platforms for tissue engineering and regenerative medicine applications [2,3]. Moreover, their easy mechanical and biological tunability and their potential responsiveness to external stimuli also permit their wide exploitation as smart drug/cell delivery carriers [4,5]. Therefore, during the last few decades, the research community has devoted great effort towards the engineering of hydrogels able to fulfil the specific requirements identified for their final application.

The mainly adopted classification of hydrogel systems is based on the origin of their polymeric component leading to the definition of synthetic and naturally derived hydrogels [6]. Such different composition strongly affects system behavior: synthetic hydrogels, such as poly(ethylene glycol) (PEG), poly(vinyl alcohol) (PVA), and poly(acrylamide) (PAA)-based formulations, are known to be characterized by strong and tunable mechanical properties and prolonged stability in aqueous media [7,8,9]. Conversely, systems of natural origin, such as gelatin, chitosan, hyaluronic acid, alginate, and peptide-based formulations, generally show enhanced bioactive features, such as biocompatibility, higher mimesis of the extracellular matrix structure, and capability to promote tissue regeneration and angiogenesis [10,11,12,13,14]. However, both synthetic and natural polymers show some drawbacks, e.g., reduced biocompatibility and weak mechanical properties and high batch-to-batch variability, respectively. Hence, to maximize their performances as biomedical devices, hydrogels should simultaneously possess the features proper of both types of systems. As a consequence, the engineering of bioartificial hydrogels, i.e., formulations containing polymers of synthetic and natural origin, has attracted great attention over the years, allowing the coupling of the key pros of both types of polymers into a single formulation [15,16,17].

Hydrogel injectability is another extremely important feature leading to mini-invasive systems able to minimize patient’s discomfort during their administration for cell or drug delivery [18,19,20]. The main requirement behind such feature lies in the capability to control the mechanism governing system transition from a viscous fluid to a solid gel. Indeed, systems should have sufficiently low viscosity to pass through small needles, but then they should quickly undergo gelation to avoid undesired dispersion of payload and polymeric chains in the surrounding tissues. Several strategies have been implemented to tune gelling kinetics over the years. Among them, great attention has been paid to host–guest [21] and electrostatic [22] interactions, Schiff-base chemistry [23], hydrogen bonding [24], disulfide bonds [25,26] and reversible Diels–Alder reactions [27,28]. However, critical cytocompatibility issues arise in the case of cell-loaded hydrogel formulations, due to frequent use of potentially toxic cross-linking agents and reactions interfering with molecules exposed on cell surfaces or the use of high-intensity UV or visible light irradiation.

**Figure 1:**
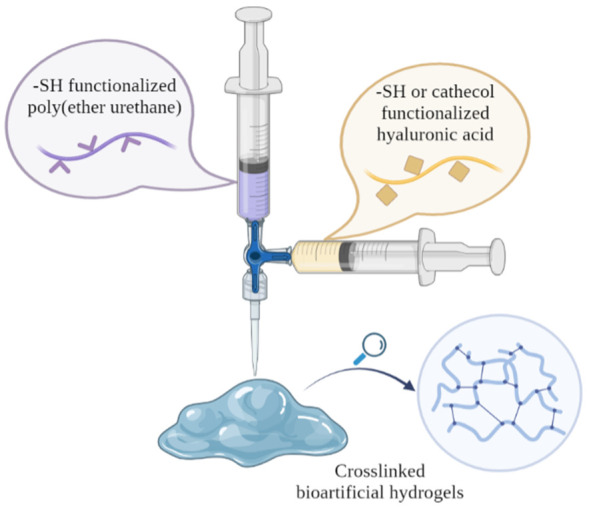
Schematic representation of the main concept of the work. Bioartificial hydrogels obtained by mixing a thiol- or catechol-modified hyaluronic acid with a thiol-modified poly(ether urethane) (PEU) through a luer-lock double syringe system. PEU amphiphilicity ensured system thermoresponsiveness and easy injectability, while grafted functional groups allowed fast in situ crosslinking.

In this scenario, this work aimed at engineering a platform of injectable bioartificial formulations by combining synthetic and naturally derived polymers properly selected to undergo in situ cross-linking upon injection (Figure 1). In more detail, the versatility of poly(urethane) chemistry was exploited to synthesize an amphiphilic poly(ether urethane) (PEU) able to provide hydrogel formulations with temperature-controlled viscosity changes [29,30]. Low molecular weight hyaluronic acid (HA) was selected due to its cytocompatibility and bioactive properties, such as the capability to promote damaged tissue regeneration through a CD44-mediated mechanism [31] and induce vascularization [32]. Moreover, its strong water retention capability makes this polymer highly suitable for aesthetic medicine applications as dermal filler being able to reduce dehydration, atrophy, and loss of elasticity [33]. To further expand the promising properties of the resultant systems, both polymers were chemically modified to expose reactive functional groups (i.e., thiol and catechol functional moieties) enabling the design of chemical hydrogels. Specifically, green and eco-friendly water-based carbodiimide chemistry was used for polymer functionalization to reduce the environmental impact. Thiol and catechol functional groups were selected to confer the resulting bioartificial formulations the ability to cross-link in physiological conditions, avoiding the need of chemical cross-linking agents and prolonging hydrogel stability in a watery environment upon injection. In this regard, we also investigated the effect of hydrogen peroxide and horseradish peroxidase addition to polymer solutions as oxidizing agent and catalyst, respectively, with the aim of improving gelation kinetics.

## 2. Results and Discussion

### 2.1. Chemical Characterization of Bioartificial Hydrogel Constituents

Before the investigation of bioartificial hydrogel preparation, catechol and thiol functionalized HA polymers were first chemically characterized to: (i) assess the success of the carbodiimide-mediated functionalization procedure, (ii) study the influence of reaction pH on the grafting yield, and (iii) quantify the number of exposed reactive moieties. Catechol- and thiol-functionalized HA polymers will be referred to with the acronyms HA-DH_pHX and HA-SH_pHX, respectively, where X indicates the pH adopted during the grafting reaction. A similar approach was also exploited for the characterization of the synthetic polymer modification. Notably, the PEU used in this work (acronym S-DHP407) was synthesized following a previously reported optimization procedure [34]. Hence, in this work only the main results are briefly reported, while for an in-depth discussion on S-DHP407 synthesis and optimization, the reader may refer to Laurano et al. [34].

#### 2.1.1. Chemical Characterization of Dopamine-Functionalized Hyaluronic Acid

**Figure 2:**
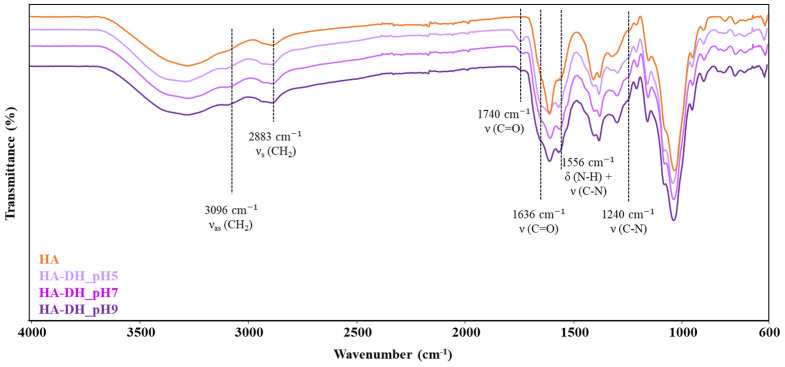
ATR-FTIR spectra of HA (orange), HA-DH_pH5 (light violet), HA-DH_pH7 (violet) and HA-DH_pH9 (dark violet). Black lines identify the wavenumber values in which HA-DH_pHX spectra exhibit major differences compared to HA spectrum (control).

The success of HA functionalization with dopamine hydrochloride (DH) molecules was first assessed through attenuated total reflectance Fourier-transform infrared (ATR-FTIR) spectroscopic analyses by comparing the spectra of HA-DH_pHX and HA control samples (Figure 2). Irrespective of the pH adopted for the grafting step, HA-DH_pHX spectra showed the appearance of the characteristic peaks ascribed to newly formed amide bonds between the carboxylic acid groups exposed along HA polymeric chains and the -NH_2_ groups of DH molecules [35,36]. The peaks at 1740 cm^−1^ and 1636 cm^−1^ can be attributed to the stretching vibration of the C=O of the amide bonds involved and not involved in H-bond interactions, respectively; the band at 1556 cm^−1^ can be ascribed to the bending and stretching of N-H and C-N bonds, respectively; the peak at 1240 cm^−1^ is produced by the stretching vibration of C-N bonds. Furthermore, the increased intensity registered at 3096 cm^−1^ and 2883 cm^−1^ in HA-DH_pHX spectra compared to the HA spectrum can be associated with the stretching vibration of the CH_2_ groups, thus further confirming the presence of DH molecules grafted to HA polymeric backbone. However, ATR-FTIR analyses did not provide clear information on the influence of reaction pH on the grafting yield, as no remarkable differences were detected among HA-DH-pHX spectra.

**Figure 3:**
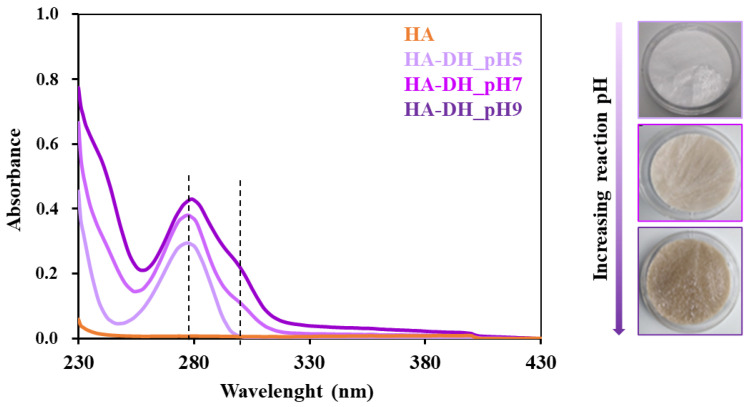
UV-vis profiles of HA (orange), HA-DH_pH5 (light violet), HA-DH_pH7 (violet) and HA-DH_pH9 (dark violet) acquired in the 230–430 nm spectral range. Dashed lines identify the peak at 280 nm exploited for DH quantification and the shoulder at 310 nm attributed to DH molecules in their oxidized form. Representative images of lyophilized HA-DH_pH5, HA-DH_pH7 and HA-DH_pH9 (from top to bottom) showing a progressively increasing brownish color.

To further investigate whether the adopted reaction pH was effectively able to tune the grafting yield of DH to HA chains, UV-vis spectroscopic analyses were conducted on HA-DH_pHX solutions and HA as reference by exploiting the characteristic absorbance at 280 nm wavelength ascribed to the aromatic ring present in the DH molecular structure. Indeed, as reported in Figure 3, in all HA-DH_pHX profiles, a strong absorbance increase was observed at 280 nm compared to the native HA, thus further proving the success of the functionalization. Moreover, remarkable differences were also observed among HA samples functionalized at different pH values: absorbance intensity at 280 nm increased for solutions of HA functionalized at increasing pH values (from HA-DH_pH5 to HA-DH_pH9). By referring to a calibration curve based on DH standards, the amount of grafted DH/g of polymer was measured to be 2.0 × 10^20^, 2.5 × 10^20^, 3.1 × 10^20^ at pH 5, 7 and 9, respectively, in accordance with carbodiimide chemistry theory. However, HA-DH_pH7 and HA-DH_pH9 profiles also showed a shoulder at 295–310 nm wavelength, attributable to the oxidation of DH molecules at alkaline pH [37]. This hypothesis was further supported by visual observations of lyophilized samples that showed more intense brownish color upon progressively raising the grafting pH from acidic to alkaline values (Figure 3).

**Figure 4:**
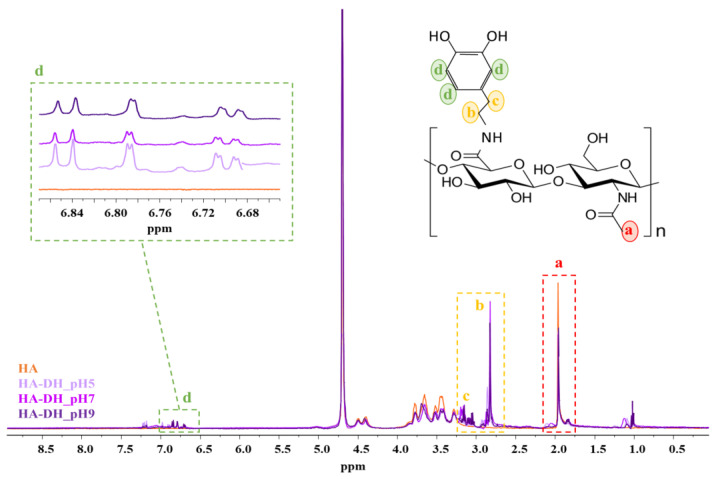
^1^H NMR spectra of HA (orange), HA-DH_pH5 (light violet), HA-DH_pH7 (violet) and HA-DH_pH9 (dark violet). Magnification in the 6.65–6.85 ppm interval highlights the appearance of the peaks ascribed to the hydrogens involved in the aromatic ring.

To definitively assess the successful HA functionalization and to quantify the amount of unoxidized DH, proton nuclear magnetic resonance (^1^H NMR) spectroscopy was performed on HA-DH_pHX samples and HA (Figure 4). Irrespective of the reaction pH, the comparison between HA-DH_pHX and HA spectra proved the carbodiimide-mediated grafting of DH molecules through the appearance of the peaks at approximately 2.80 ppm, 3.15 ppm and 6.65–6.85 ppm, ascribed to the hydrogens of the aliphatic chain and to the hydrogens involved in the aromatic ring of catechol groups in unoxidized DH, respectively [38]. The signal in the chemical shift range between 6.65 and 6.85 ppm was thus used to quantify the amount of unoxidized catechol groups exposed along the HA backbone by varying the pH of the grafting phase from 5 to 9. By referring to the DH molecule spectrum, the number of grafted DH in the unoxidized form per gram of polymer was measured to be 1.9 × 10^20^, 5.1 × 10^19^ and 6.1 × 10^19^ for HA-DH_pH5, HA-DH_pH7 and HA-DH_pH9, respectively. The performed quantification revealed a decreasing number of catechol groups with increasing the grafting pH from 5 to 7. These results were in agreement with UV-vis analysis, showing the appearance of an absorption peak as a shoulder in HA-DH_pH7 UV-vis spectrum respect to that of HA-DH_pH5 (Figure 3). Hence, they further demonstrated the occurrence of pH-induced catechol oxidation into quinones. Conversely, in the case of HA-DH_pH9, the amount of not oxidized DH molecules turned out to be slightly higher than HA-DH_pH7. This discrepancy can be explained by the total amount of grafted DH molecules being higher at alkaline than neutral pH values, as also suggested by UV-vis analysis. Table 1 reports the number of oxidized and unoxidized DH molecules as calculated by comparing the results obtained from UV-vis and ^1^H NMR spectroscopic analyses.

As a matter of fact, by changing the pH of the grafting reaction, it was possible to tune the yield of HA functionalization with DH molecules. Specifically, the highest amount of grafted DH was obtained by setting an alkaline pH (i.e., pH 9) in accordance with the theory of carbodiimide chemistry. However, due to the high reactivity of catechol groups, which easily undergo oxidation into quinones at alkaline pH [39], pH 5 was selected as the optimal condition. At this pH value, grafting of approximately 10^20^ DH molecules per gram of polymer was obtained, with no significant oxidation phenomena. Hence, HA functionalized at pH 5 was further investigated for bioartificial hydrogel formulation.

#### 2.1.2. Chemical Characterization of Cysteine-Functionalized Hyaluronic Acid

**Figure 5:**
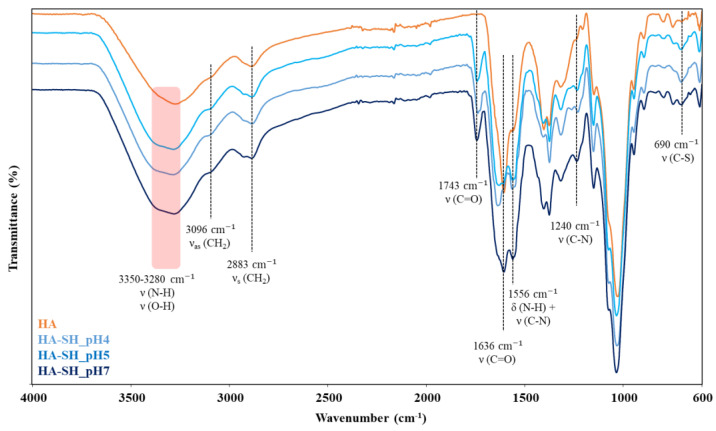
ATR-FTIR spectra of HA (orange), HA-SH_pH4 (light blue), HA-SH_pH5 (blue) and HA-SH_pH7 (dark blue). Black lines and the red color band identify the spectral regions in which HA-SH_pHX samples exhibit major differences compared to HA (control).

Similarly to HA-DH_pHX, the as-synthesized HA-SH_pHX samples were characterized by ATR-FTIR spectroscopy to assess the successful grafting of L-cysteine methyl ester hydrochloride (Cys) to the HA backbone through amide bonds. Figure 5 compares the ATR-FTIR spectra of HA, HA-SH_pH4, HA-SH_pH5 and HA-SH_pH7. The spectra suggested the success of the functionalization procedure [40]. Indeed, bands ascribed to amide bonds appeared in HA-SH_pHX spectra, i.e., the stretching of N-H and C-N bonds at 1630 cm^−1^ and 1240 cm^−1^, respectively, and C-N stretching and N-H bending (scissoring mode) vibrations at 1556 cm^−1^. Moreover, an increase in the intensity of the signals in the wavenumber range between 3280 and 3350 cm^−1^ was observed, due to the stretching vibration of N-H bonds. Similarly, the increased intensity of the peak at 690 cm^−1^ was due to the stretching vibration of C-S linkages. Lastly, a new absorption band appeared at 1743 cm^−1^ due to the stretching vibration of the carbonyl bonds present in the grafted Cys, as proved by the comparison among the ATR-FTIR spectra of Cys and HA-SH_pHX samples (data not reported). Conversely to HA-DH_pHX samples, in the ATR-FTIR spectra of HA-SH_pHX, the amide bond signal intensity increased with increasing the pH adopted during the grafting phase. According to the theoretical mechanism of carbodiimide-mediated chemistry, the degree of grafting increased with increasing the grafting pH from 4 to 7. However, the adoption of higher pH values during the grafting phase is expected to favor thiol group oxidation, leading to disulfide bond formation [25,26,41]. Apart from this, the exposed free -SH groups were protected from oxidation by performing the dialysis procedure required to wash out residual Cys and reaction by-products using a dialysis medium at acidic pH (i.e., pH 4). The collected freeze-dried material was stored at 4 °C under nitrogen atmosphere, according to optimized storage conditions we have defined for poly(ether urethane) thiomers in our previous work [34]. Nevertheless, no clear evidence of the effective exposure of -SH groups along HA chains could be gathered from the analysis of ATR-FTIR spectra. Indeed, no bands attributed to S-H bond vibrations could be clearly identified, due to the characteristic low intensity of the S-H stretching band within 2600 and 2550 cm^−1^ and the broad signal produced by -CH_2_, N-H and -OH stretching between 2300 and 3700 cm^−1^.

In this work the effective exposure of free -SH functionalities along HA-SH_pHX was colorimetrically assessed by Ellman’s test. Ellman’s colorimetric assay is a well-established approach that definitively and easily proves the presence of free thiol groups within a sample through a sample color change from transparent to yellowish [42,43]. Such color transition results from the reaction between the thiol groups present within the samples and Ellman’s reagent (i.e., 5,5′-dithio-bis-2-nitrobenzoic acid or DTNB) that produces a yellowish product. By measuring the absorbance of these yellow reaction products and through a properly constructed calibration curve, Ellman’s colorimetric assay also allows the quantification of the amount of free thiol groups present within the analyzed samples.

The visual inspection of the samples upon incubation with Ellman’s reagent immediately proved the successful exposure of -SH groups along the HA backbone, as highlighted in Figure S1a, which shows as an example a photo of HA (control) and HA-SH_pH5 samples. UV-vis analysis of HA and HA-SH_pHX samples after Ellman’s test allowed the estimation of the number of exposed -SH groups per gram of polymer (Figure S1b). Although HA-SH_pH7 showed the highest functionalization yield in ATR-FTIR spectroscopic analyses, the Ellman’s colorimetric assay performed on this material quantified a number of exposed thiol groups (i.e., 1.0 × 10^20^ -SH/g) to be significantly lower than HA-SH_pH5 (i.e., 1.9 × 10^20^ -SH/g, *p* = 0.0074). This result is in agreement with the expected increased thiol oxidation with increasing grafting reaction pH. Conversely, in the case of HA-SH_pH4, the number of exposed -SH groups/g of polymer (i.e., 0.9 × 10^20^ -SH/g) was significantly lower with respect to both HA-SH_pH5 and HA-SH_pH7 (*p* = 0.0008 and 0.0036, respectively), in agreement with the more unfavorable pH adopted to graft Cys to the HA polymer chains. Overall, a pH value of 5 turned out to maximize the number of free thiol groups exposed along thiolated-HA polymeric chains, representing the best compromise to maximize the bulk functionalization degree of HA with Cys through the carbodiimide chemistry while minimizing the oxidation of the exposed sulfhydryl moieties. The optimized protocol for the thiolation of HA polymeric chains through carbodiimide chemistry is in agreement with the reaction conditions adopted to synthesize the thiol-grafted poly(ether urethane) that has already been optimized by our group [34].

#### 2.1.3. Chemical Characterization of Thiol-Grafted Poly(Ether Urethane)

The synthesis of the thiol-grafted poly(ether urethane) with the acronym S-DHP407 was assessed step by step through chemical characterizations and colorimetric assays. A detailed analysis of the results of these tests can be found in our previous work [34]. Briefly, size-exclusion chromatography (SEC) coupled with ATR-FTIR and ^1^H NMR spectroscopy proved the successful synthesis of a high molecular weight P407-based poly(ether urethane) (DHP407, number average molecular weight of 33–35 kDa, polydispersity index of around 1.6). The same characterizations were performed on the Boc-deprotected poly(ether urethane) D-DHP407, demonstrating the complete absence of any chemical degradation induced by the performed deprotection reaction and the successful exposure of secondary amines along its polymeric chains (around 4.5 × 10^20^ -NH functionalities/g of D-DHP407 were quantified through the Orange II sodium salt colorimetric assay). Lastly, the successful carbodiimide-mediated bulk functionalization of D-DHP407 with thioglycolic acid (TGA) molecules resulting in S-DHP407 poly(ether urethane) was assessed by ATR-FTIR, ^1^H and ^13^C NMR spectroscopy, which evidenced the typical peaks of the newly formed amide bonds and proved the exposure of free -SH moieties along the polymeric backbone. The amount of exposed sulfhydryl groups along S-DHP407 chains was estimated through the Ellman’s colorimetric assay and turned out to be approximately 1.7 × 10^19^ per g of PEU.

### 2.2. Preliminary Evaluation of Poly(Ether Urethane)-Hyaluronic Acid Miscibility and Injectability

**Figure 6:**
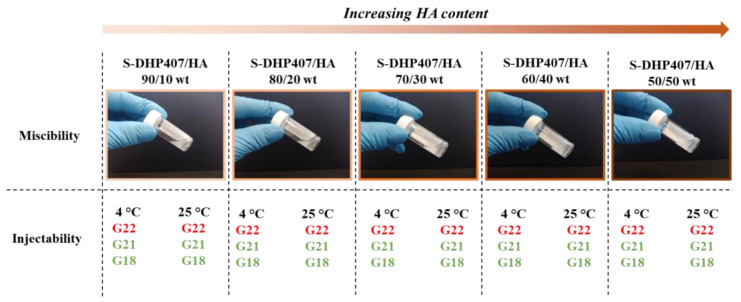
Representative photos of S-DHP407/HA formulations with increasing HA content from left to right (overall polymeric concentration 15% *w*/*v*) proving the successful miscibility of the synthetic and natural polymers at the macroscale. Injectability assessment at two temperatures (i.e., 4 °C and 25 °C) through G18, G21 and G22 needles. Green and red stand for assessed and unassessed injectability, respectively.

To evaluate the molecular compatibility of the selected synthetic and natural components, preliminary miscibility tests were conducted by blending S-DHP407 PEU and HA before further functionalization with thiol or catechol reactive groups. Specifically, different formulations were prepared at 15% *w*/*v* polymeric concentration and S-DHP407/HA weight ratio ranging between 90/10 and 50/50. As shown in Figure 6, irrespective of the tested condition, all the systems appeared slightly turbid, this being a feature of the synthetic counterpart. No phase-separation phenomena were observed upon sample incubation at 4 °C or 37 °C for 24 h. Hence, despite the presence of a temperature-controlled gelling component (i.e., S-DHP407) able to undergo self-assembly at 37 °C inducing phase separation, this visual inspection at the macroscale suggested polymer miscibility, irrespective of the tested temperature. To preliminarily investigate their potential exploitation as mini-invasive systems for biomedical or dermatological applications, injectability tests were performed through needles of different size (i.e., G18, G21 and G22) at 4 °C and 25 °C, as temperature is a potentially critical parameter affecting system viscosity. Indeed, low temperatures should enhance injectability of the synthetic component, as S-DHP407 aqueous solutions with concentration from 9% *w*/*v* to 25% *w*/*v* are in the sol state at 4 °C and in a gel state at 37 °C [30,34]. Conversely, high temperatures should improve the injectability of the natural counterpart by lowering its viscosity. Nevertheless, all formulations turned out to be injectable through G21 and G18 needles, both at 4 °C and 25 °C, as assessed by three independent users. However, systems could not be injected through G22 needles.

To further investigate polymer miscibility at the microscale, scanning electron microscopy (SEM) images were acquired on lyophilized S-DHP407, S-DHP407/HA and HA samples subjected to a controlled freezing procedure. As an example, Figure S2 shows SEM images of the polymers and their blend at 50/50 *w*/*w* (i.e., the formulation with the highest HA content). Such images qualitatively confirmed the formation of a homogeneous network with no evident HA or S-DHP407 phase separation.

**Figure 7:**
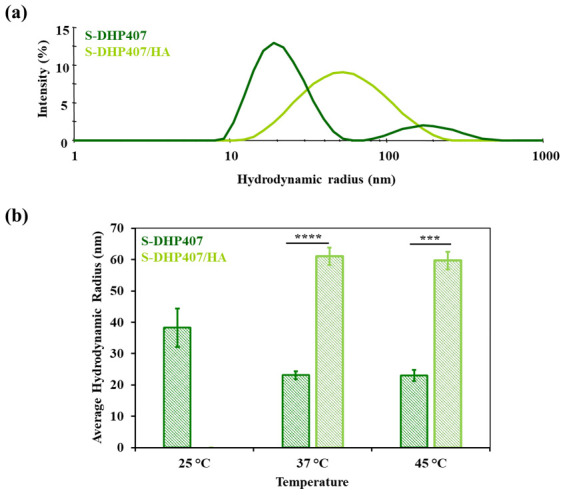
(**a**) Representative hydrodynamic radius vs. intensity profiles acquired for S-DHP407 (dark green) and S-DHP407/HA 50/50 *w*/*w* (light green) at 37 °C. (**b**) Average hydrodynamic radius calculated for S-DHP407 (dark green) and S-DHP407/HA 50/50 *w*/*w* (light green) formulations at 25 °C, 37 °C and 45 °C. (*** *p* = 0.0004 and **** *p* < 0.0001).

To further investigate polymeric chain interactions at the nanoscale, dynamic light scattering (DLS) analysis was performed on S-DHP407, HA and S-DHP407/HA_50/50 *w*/*w* solutions at 25 °C, 37 °C and 45 °C. Figure 7a,b shows the hydrodynamic radius intensity pattern at 37 °C and the average hydrodynamic radius of the formulations at 25 °C, 37 °C and 45 °C, respectively. Figure 7a shows a double peak profile for S-DHP407 solution at 37 °C, in agreement with previous findings [34,42]. In detail, the more intense peak centered at approximately 20 nm was attributed to micellar spherical structures, while the peak with lower intensity at approximately 200 nm was ascribed to the presence of micellar aggregates. S-DHP407 capability to form single micelles and clusters at 37 °C can be attributed to the presence of amphiphilic Poloxamer^®^ 407 (P407) copolymer as one of the block constituents of the PEU [29]. Conversely, the DLS spectrum of HA solution suggested the lack of chain organization into spherical systems at any tested temperature (All data are available upon request from the corresponding author). The DLS spectrum of S-DHP407/HA_50/50 *w*/*w* blend solution showed the presence of a single peak centered at higher values (around 60 nm) compared to S-DHP407 solution alone, suggesting interactions between S-DHP407 and HA chains, with the formation of bigger aggregates. Moreover, although HA is not a thermoresponsive polymer, its presence in the mixture did not interfere with S-DHP407 chain capability to arrange into organized structures upon temperature increase.

The average hydrodynamic radius of S-DHP407 micelles (Figure 7b) decreased as a function of temperature increase from 25 °C to 45 °C, as previously observed in the case of similar P407-based PEUs [34,44]. Such behavior was attributed to a temperature-driven process of chain organization into micelles. By further increasing temperature, micelles packed into a tighter network, with the removal of water molecules from the hydrophobic micelle core towards interstitial spaces. In blend solution, no well-defined interactions were observed between S-DHP407 and HA polymeric chains at 25 °C due to the high variability in the measured hydrodynamic radius, probably due to the presence of HA, which slightly hindered chain arrangement into micelles. Conversely, at 37 °C and 45 °C, spherical structures with higher average hydrodynamic radius than for S-DHP407 formed (*p* < 0.0001 and *p* = 0.0004, respectively). Such findings suggested the establishment of strong interactions between S-DHP407 and HA chains, probably through hydrogen bonding [45]. Moreover, the significant increase in average hydrodynamic radius of S-DHP407/HA 50/50 *w*/*w* respect to S-DHP407 formulation could be attributed to the formation of a hydrophilic HA-based shell on S-DHP407 hydrophilic micelle tails [46]. Lastly, DLS analysis demonstrated that the addition of a non-thermosensitive component such as HA did not alter S-DHP407 thermo-responsiveness in S-DHP407/HA_50/50 *w*/*w* blend solution.

### 2.3. Bioartificial Hydrogel Characterization

S-DHP407-based bioartificial hydrogel formulations were prepared using HA-DH_pH5 or HA-SH_pH5 based on results on HA functionalization, previously discussed. pH 5 turned out to be the optimal grafting condition to maximize the success of the reaction while preserving thiol and catechol group functionality. Hereafter, HA-DH_pH5 and HA-SH_pH5 will be referred to as HA-DH and HA-SH, respectively. Irrespective of their composition, bioartificial hydrogels were prepared at 15% *w*/*v* polymeric concentration, 50/50 *w*/*w* ratio between the natural and the synthetic counterpart and 1:1 functional group molar ratio. Gelation potential in physiological conditions and stability in aqueous environment at 37 °C were evaluated in the as-prepared blends and upon addition of H_2_O_2_ (1:1 molar ratio with respect to HA functional groups) and horseradish peroxidase (HRP, 1:1 and 2:1 molar ratio with respect to HA thiol/catechol groups), aiming to study the effects of oxidizing agent addition on hydrogel gelation kinetics [47,48,49]. Table 2 summarizes the nomenclature adopted to refer to all the designed hydrogel formulations, while Figure S3 and Video S1 show the double-syringe system used for their mixing.

#### 2.3.1. Hydrogel Gelation at Physiological Temperature

**Figure 8:**
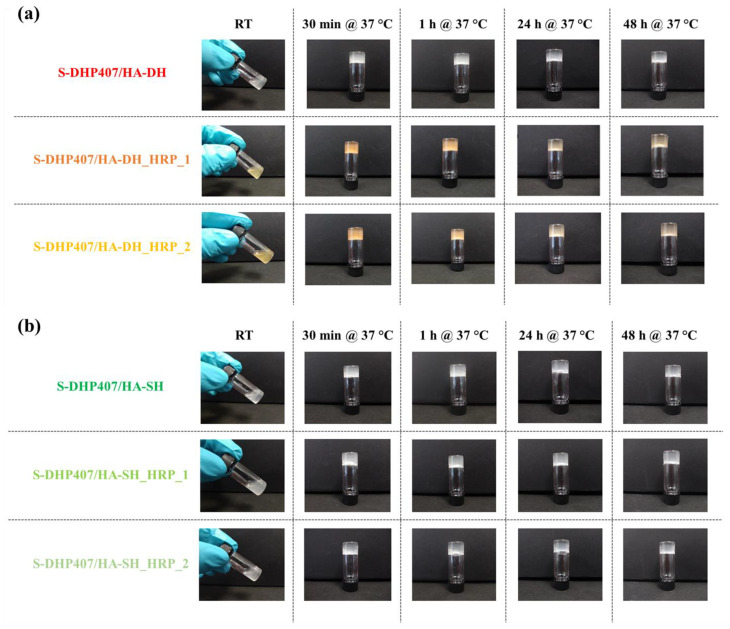
Representative photos of the tube-inverting test applied to (**a**) S-DHP407/HA-DH, S-DHP407/HA-DH_HRP_1 and S-DHP407/HA-DH_HRP_2, and (**b**) S-DHP407/HA-SH, S-DHP407/HA-SH_HRP_1 and S-DHP407/HA-SH_HRP_2 bioartificial samples at different temperatures and time points: immediately after preparation at room temperature (RT) and at 30 min, 1 h, 24 h and 48 h incubation at 37 °C.

The capability of the developed bioartificial hydrogels to undergo a sol-to-gel transition in physiological conditions was tested through the tube-inverting test. To this end, the samples (1 mL) were equilibrated at 37 °C in an incubator and inverted at predefined time points to assess their sol or gel state by checking the presence or absence of flow along the vial walls, respectively. Figure 8a,b reports the images of the different formulations taken after 30 min, 1 h, 1 day and 2 days of incubation at 37 °C. In the same figures, the images of the samples immediately after preparation at room temperature (RT) are also reported, evidencing that under these experimental conditions, all the bioartificial hydrogels were in the sol state.

Upon incubation at physiological temperature for 30 min, bioartificial samples containing either HA-DH or HA-SH appeared in the gel state, as assessed by the absence of flow along the vial walls during the 30 s inversion time. Furthermore, the achieved gel state at 37 °C persisted over time up to 2 days of observation. The use of HA-DH or HA-SH as natural counterpart of the bioartificial hydrogels did not evidence clear differences in the gelation potential of the resulting formulations. Similarly, the addition of H_2_O_2_ and HRP (at two different molar ratios with respect to HA thiol and catechol groups) did not affect the sol-to-gel transition time at 37 °C. Indeed, the tube-inverting test showed that after 10 and 20 min incubation at 37 °C, samples were in a sol state, irrespective of their composition (data not reported).

S-DHP407/HA-DH blend samples showed a pearl-gray color within the whole observation window (Figure 8a), while the addition of H_2_O_2_ and HRP in S-DHP407/HA-DH_HRP_1 and S-DHP407/HA-DH_HRP_2 induced a color change from pearl gray to yellowish immediately after hydrogel preparation at 25 °C. The samples progressively shifted from yellowish to orange color up to 1 h observation, as a consequence of the increasing number of catechol groups undergoing oxidation and forming quinones [50]. From 2 h incubation time, the color of the gels started to bleach, becoming light yellow after 2 days of storage at physiological temperature.

Conversely, S-DHP407/HA-SH samples (Figure 8b) exhibited a pearl-gray color irrespective of their composition and did not show any color transition within 2 days observation at 37 °C.

Hence, the tube-inverting test evidenced the capability of hydrogels to undergo sol-to-gel transition within 30 min at physiological temperature and to keep this status over time, but no clear evidence of the effective formation of chemical gels was obtained.

**Figure 9:**
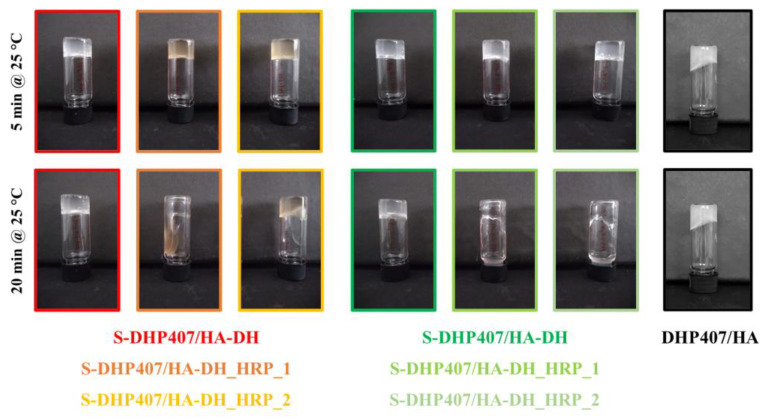
Representative photos of bioartificial S-DHP407/HA-DH and S-DHP407/HA-SH formulations with and without H_2_O_2_ and HRP showing their sol or gel state upon equilibration at 25 °C for 5 and 20 min. DHP407/HA system was also monitored as control condition.

A DHP407/HA control sample also exhibited gelation after 30 min of incubation at 37 °C due to the thermoresponsive behavior provided by the P407-containing PEU counterpart, as observed at the nanoscale from DLS analyses (data not reported). To indirectly verify the formation of covalent bonds between S-DHP407 and functionalized HA chains, after 2 days of storage at 37 °C, the samples were again equilibrated at 25 °C and their sol/gel state assessed by inverting the vials after 5 and 20 min (Figure 9). After 5 min at 25 °C, all formulations retained a gel state, with the exception of control physical hydrogel based on DHP407 and HA. This observation clearly proved that covalent bonds effectively formed between S-DHP407 and thiol- or dopamine-grafted HA chains. However, after 20 min of equilibration at 25 °C, only S-DHP407/HA-SH and S-DHP407/HA-DH samples still appeared in the gel state, while all the samples containing H_2_O_2_ and HRP underwent gel-to-sol transition. This behavior could be ascribed to the addition of hydrogen peroxide: although it was added at 1:1 molar ratio with respect to the thiol or catechol groups exposed along HA chains, it partially exerted its oxidizing effect on polymers inducing their partial degradation [51,52]. Chemical oxidation/degradation phenomena of polymers did not affect the formation of hydrogels at 37 °C, as a result of physical and chemical interactions among the polymer chains. However, the formed gel network was not stable at 25 °C, probably because partial polymer degradation occurred, making the hydrogel mesh defective and poorly stable at 25 °C, a temperature at which physical interactions among poly(urethane) micelles also tend to decrease.

Overall, the characterizations suggested the effective formation of chemical hydrogels based on S-DHP407 and functionalized HA, but highlighted that the addition of H_2_O_2_ and HRP did not improve gel formation kinetics due to the occurrence of polymer degradation phenomena that prevailed over the expected beneficial effects of oxidation of HA and S-DHP407 functional groups in enhancing cross-linking.

#### 2.3.2. Hydrogel Stability in Physiological Mimicking Conditions

**Figure 10:**
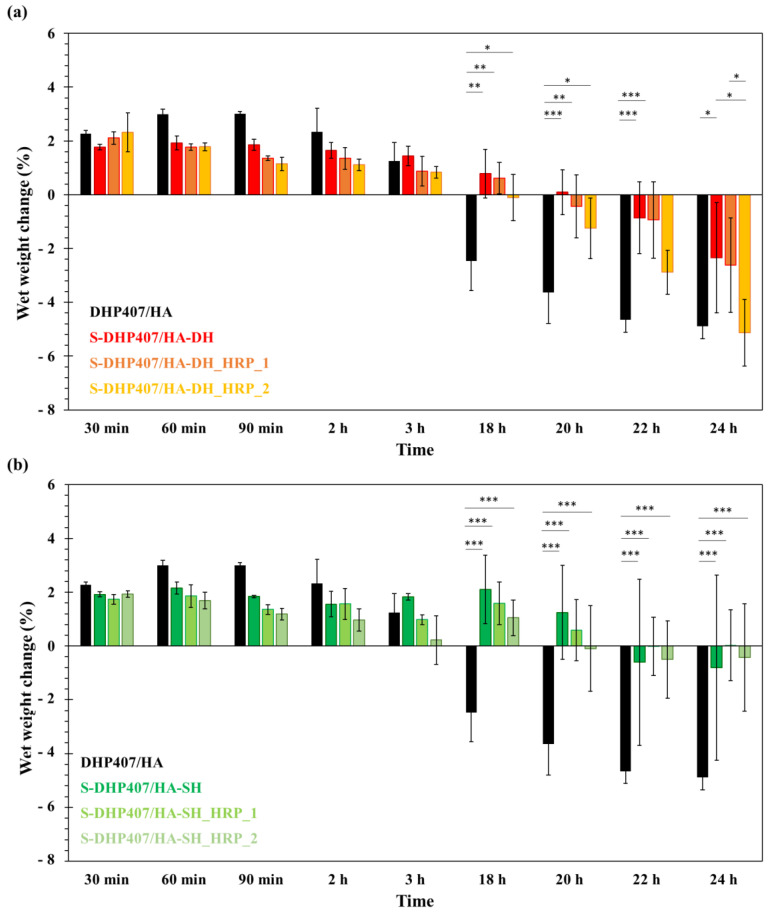
Percentage of wet weight change as measured for the developed bioartificial formulations and control samples during incubation in contact with PBS (pH 7.4) at 37 °C. (**a**) Stability of DHP407/HA (black) and HA-DH-based blends with S-DHP407 (i.e., S-DHP407/HA-DH, S-DHP407/HA-DH_HRP_1 and S-DHP407/HA-DH_HRP_2); (**b**) Stability of DHP407/HA (black) and HA-SH-based blends with S-DHP407 (i.e., S-DHP407/HA-SH, S-DHP407/HA-SH_HRP_1 and S-DHP407/HA-SH_HRP_2). (* *p* < 0.05, ** *p* < 0.02, *** *p* < 0.001).

The in vitro testing of hydrogel swelling/stability in physiological mimicking conditions is of crucial importance to predict the in vivo behavior of the systems and thus their potential applicability. Therefore, in this work, a preliminary assessment of bioartificial formulation stability in a watery environment was conducted up to 24 h. Specifically, the gels were put in contact with PBS at 37 °C and the variation in their wet weight was monitored at predefined time intervals. As control condition, purely physical gels based on a blend of DHP407 and HA were also tested. Figure 10 reports the percentage of wet weight change measured for S-DHP407 blends with HA-DH (Figure 10a) or HA-SH (Figure 10b) and control samples. DHP407/HA gels showed the typical behavior of purely PEU-based thermosensitive hydrogels [53,54]. Their wet weight initially increased due to the absorption of PBS from the surrounding environment. Then, from 3 h incubation time, the percentage of wet weight change showed a decrease, becoming negative from 18 h on. This behavior suggested that the samples also released their polymeric components while absorbing PBS, with prevalent dissolution over swelling from 18 h incubation. With regard to the chemically cross-linked samples, in general they showed a similar trend to DHP407/HA gels, but the complete preeminence of dissolution/erosion over PBS absorption was delayed, with wet weight change becoming negative only after around 1 day of incubation. Indeed, at both 18 h and 20 h observation time, the wet weight change of DHP407/HA physical gels was significantly higher in absolute value compared to S-DHP407/HA-DH (*p* < 0.01 and *p* < 0.001 at 18 h and 20 h, respectively), S-DHP407/HA-DH_HRP_1 (*p* < 0.01 at both 18 h and 20 h) and S-DHP407/HA-DH_HRP_2 (*p* < 0.05 at both 18 h and 20 h). Conversely, in the case of bioartificial hydrogels based on HA-SH, control samples showed higher wet weight change in absolute value than all HA-SH-based bioartificial samples starting from 18 h incubation (*p* < 0.001 at each time point). Among the three HA-DH-based bioartificial gels investigated in this work, S-DHP407/HA-DH_HRP_2 samples exhibited a more similar behavior to DHP407/HA physical gels, probably because of their higher HRP content, catalyzing polymer oxidative degradation beside catechol oxidation and reaction with thiol groups. This behavior was not observed in S-DHP407/HA-SH_HRP_2 gels that exhibited higher stability compared to DHP407/HA physical gels, with significant differences at 18, 20, 22 and 24 h observation. Although polymers in S-DHP407/HA-SH_HRP_2 samples probably underwent chemical degradation (as hypothesized based on results from the tube inverting test), the resulting gel network was probably better developed than S-DHP407/HA-DH_HRP_2 due to the faster curing kinetics of thiol–thiol chemistry compared to catechol–thiol one [55]. This difference also explains the overall higher stability of HA-SH-based formulations compared to HA-DH-containing ones, although significant differences only appeared for prolonged incubation times (S-DHP407/HA-DH vs. S-DHP407/HA-SH no significant differences, S-DHP407/HA-DH_HRP_1 vs. S-DHP407/HA-SH_HRP_1 significant difference at 24 h incubation time (*p* < 0.05), S-DHP407/HA-DH_HRP_2 vs. S-DHP407/HA-SH_HRP_2 significant difference at 22 h (*p* < 0.05) and 24 h (*p* < 0.001) incubation times). Among HA-DH-based hydrogels, S-DHP407/HA-DH gels showed the best stability in watery media, showing a significantly lower wet weight change compared to S-DHP407/HA-DH_HRP_2 and DHP407/HA after 24 h incubation with PBS (*p* < 0.05). Similarly, within the tested HA-SH-based formulations, S-DHP407/HA-SH gels exhibited the highest stability, although no significant differences were observed compared to S-DHP407/HA-SH_HRP_1 and S-DHP407/HA-SH_HRP_2. Conversely, statistically significant differences were observed between S-DHP407/HA-SH and DHP407/HA gels from 18 h incubation (*p* < 0.001). Moreover, S-DHP407/HA-DH and S-DHP407/HA-SH also exhibited a lower degree of swelling compared to DHP407/HA control gels because of the formation of a chemically cross-linked gel network, as also proved by the tube-inverting test results. For these samples, dissolution/erosion prevalence over PBS absorption appeared from 18–20 h incubation time, whereas all other samples showed a first decrease in their wet weight in the time interval between 90 min and 3 h of incubation with PBS at 37 °C. Lastly, S-DHP407/HA-DH_HRP_1 and S-DHP407/HA-SH_HRP_1 showed an intermediate behavior between S-DHP407/functionalized-HA samples (i.e., S-DHP407/HA-DH and S-DHP407/HA-SH) and gels containing HRP at 2:1 molar ratio with respect to HA thiol/catechol groups (i.e., S-DHP407/HA-DH_HRP_2 and S-DHP407/HA-SH_HRP_2). This is in accordance with the hypothesized H_2_O_2_-induced oxidation of polymer chains, which for these samples most likely occurred at a lower extent due to lower HRP content and thus catalyzing effect.

## 3. Conclusions

In this work, a platform of bioartificial in situ cross-linkable injectable hydrogels based on functionalized poly(ether urethane) and hyaluronic acid was developed upon optimization of a green functionalization procedure. S-DHP407 was synthesized by grafting TGA to the backbone of a thermoresponsive P407-based poly(ether urethane) bearing -NH groups following a protocol recently optimized by our group [34]. Bulk functionalization of HA with either dopamine or cysteine (providing catechol and thiol groups, respectively) was optimized using carbodiimide chemistry. Particularly, pH 5 was selected to perform the grafting reaction, representing a compromise solution to maximize grafting yield while preserving grafted functional groups from undesired oxidation. Hydrogels were obtained by simply mixing the two polymeric components through a Luer lock double-syringe system. No evident phase separation occurred between the natural and synthetic components, as observed through qualitative miscibility tests and SEM analysis. Additionally, DLS analysis suggested the establishment of interactions between the synthetic and the natural polymer chains. The formulations could also be easily injected through needles with different diameters. Tube-inverting tests showed that all the formulations underwent a sol-to-gel transition within 30 min incubation at 37 °C. The absence of a gel-to-sol transition upon temperature decrease to 25 °C indirectly demonstrated the successful formation of covalent bonds between S-DHP407 and HA-SH or HA-DH. In physiological mimicking conditions, S-DHP407/HA-DH and S-DHP407/HA-SH formulations exhibited improved stability compared to blend hydrogel based on unfunctionalized polymers. The addition of H_2_O_2_ and HRP did not improve the gelation kinetics and decreased gel stability, probably due to PEU and HA chemical degradation. Faster curing kinetics and higher reactivity of thiol–thiol groups than thiol–catechol functionalities made HA-SH-containing bioartificial hydrogels the most stable formulations developed in this work.

## 4. Materials and Methods

### 4.1. Materials

Naturally derived hyaluronic acid (HA, ${\bar{M}}_{n}$ 90,000 Da) was purchased from Giusto Favarelli (Milan, Italy). Dopamine hydrochloride (DH), L-cysteine methyl ester hydrochloride (Cys), thioglycolic acid (TGA), N-hydroxysuccinimide (NHS), hydrogen peroxide, horseradish peroxidase (HRP, > 150 U/mg), Poloxamer^®^ 407 (P407), N-Boc diethanolamine (N-Boc), 1,6-hexamethylene diisocyanate (HDI), and dibutyltin dilaurate (DBTDL) were obtained from Merck (Milan, Italy), 1-ethyl-3-(3-dimethylaminopropyl) carbodiimide (EDC) was purchased from TCI Chemicals (Zwijndrecht, Belgium), and 1,2-dichloroethane (DCE) and all other solvents were purchased from Carlo Erba Reagents (Cornaredo, Italy). Before the poly(ether urethane) synthesis, P407, N-Boc, HDI and DCE were treated to remove residual water molecules and stabilizers according to the protocols described in [34].

### 4.2. Hyaluronic Acid Functionalization with Dopamine and L-cysteine Methyl Ester

HA functionalization with DH and Cys was performed exploiting the carbodiimide-mediated amide bond formation between -COOH and -NH_2_ groups exposed along HA and grafting molecule chains, respectively. To this purpose, EDC (100 mg/mL) and NHS (50 mg/mL) were first dissolved in double-demineralized water (ddH_2_O) at 2:1 molar ratio with respect to the theoretical number of HA-COOH groups and then added to HA previously solubilized in ddH_2_O in order to reach a final polymeric concentration equal to 1.5% *w*/*v*. The pH was then adjusted to 5 and the mixture kept under magnetic stirring at 4 °C for 1 h and in the dark to allow -COOH group activation. Meanwhile, DH or Cys solutions were prepared by dissolving the molecules in ddH_2_O at 2:1 molar ratio with respect to carboxylic acid groups and added to the activated HA solution. To optimize the grafting step, the pH was then adjusted at different values. In detail, DH grafting was carried out at pH 5, 7 or 9 for 2 h at 4 °C in the dark and under magnetic stirring. pH 4, 5 and 7 were considered for Cys grafting while keeping constant all other reaction conditions. Such grafting pH values were selected based on a previous work describing the grafting optimization of a thiol-bearing molecule to a PEU backbone aiming at maximizing the grafting yield while preserving free thiol groups [34]. Afterward, the mixture was put in dialysis (cut-off membrane 10–12 kDa, Merck, Milan, Italy) for 48 h against ddH_2_O adjusted at pH 4 with HCl 1 M to avoid functional group oxidation. Finally, the mixture was freeze-dried using a Martin Christ ALPHA 2-4 LSC and stored under nitrogen atmosphere until use.

HA functionalized with DH and Cys will be referred to with the acronyms HA-DH_pHX and HA-SH_pHX, respectively, where X stands for the adopted pH in the grafting step.

### 4.3. Chemical Characterization of Dopamine-Functionalized Hyaluronic Acid

#### 4.3.1. Attenuated Total Reflectance Fourier-Transform Infrared Spectroscopy

Attenuated total reflectance Fourier-transform infrared (ATR-FTIR) spectroscopy was conducted to assess the success of DH and Cys grafting and to investigate differences ascribed to the adopted pH in the grafting step. Analyses were performed on functionalized HA-DH_pHX and HA (as control condition) using a Spectrum 100 (PerkinElmer, Waltham, MA, USA) equipped with an ATR accessory with diamond crystal. Spectra were acquired in the range 4000–600 cm^−1^ at RT and 32 scans with 4 cm^−1^ resolution.

#### 4.3.2. UV-Vis Spectroscopy

UV-vis spectroscopic analyses were performed to quantify the amount of grafted DH to HA polymeric chains by considering the absorbance of the signal at 280 nm ascribed to the DH aromatic ring. Analyses were performed on all HA-DH_pHX and HA as such (as control condition) using a PerkinElmer Lambda 25 UV-vis spectrophotometer. Samples were prepared by dissolving the polymer in ddH_2_O at 1.7 mg/mL concentration. A calibration curve based on DH standards (concentration: 0.01–1 mM) was also prepared by dissolving the molecule in ddH_2_O and used for the quantification of the number of DH units exposed along HA chains. Spectra were acquired in the 430–230 nm spectral range at RT. Analyses were performed in triplicate and results are reported as means ± standard deviation.

#### 4.3.3. Proton Nuclear Magnetic Resonance Spectroscopy

To assess the success of HA functionalization with DH molecules and to investigate the influence of reaction pH on the grafting yield, proton nuclear magnetic resonance (^1^H NMR) spectroscopy was performed on all HA-DH_pHX and HA as reference. Grafted DH was quantified by comparing the area of the peak ascribed to the protons of the aromatic ring between 6.65 and 6.85 ppm in HA-DH_pHX spectra with the corresponding area measured for DH alone. Samples were prepared by dissolving 10 mg of polymer in 1 mL of deuterium oxide and analyzed using an Avance III Bruker spectrometer equipped with a 11.75 T superconductor magnet (500 MHz ^1^H Larmor frequency). Spectra resulted from the average of 12 runs with 10 s of relaxation time.

### 4.4. Chemical Characterization of L-cysteine-Functionalized Hyaluronic Acid

#### 4.4.1. Attenuated Total Reflectance Fourier-Transform Infrared Spectroscopy

ATR-FTIR analyses were performed on HA-SH_pHX samples and HA as control to investigate the success of the functionalization procedure and to evaluate the influence of the adopted pH in the grafting step. Samples were analyzed according to the protocol described in Section 4.3.1.

#### 4.4.2. Thiol Quantification through Ellman’s Method

The quantification of free thiol groups exposed along HA-SH_pHX chains was carried out through the Ellman’s method according to a protocol recently published by Laurano et al. [34]. Briefly, 1 mg of polymer was first dissolved in 875 µL of ddH_2_O and then added to 875 µL of dibasic sodium phosphate buffer 0.5 M adjusted at pH 8 and 1.75 mL of Ellman’s reagent (i.e., 5,5′-dithio-bis-2-nitrobenzoic acid 1.5 mM in dibasic sodium phosphate 0.5 M). Subsequently, samples were incubated for 3 h at RT and in the dark to allow the coupling reaction between Ellman’s reagent and free -SH groups, leading to the formation of yellow products. Unfunctionalized HA was subjected to the same protocol as control condition. Afterwards, sample absorbance at 412 nm was measured through an UV-vis spectrophotometer (PerkinElmer, Lambda 25) and thiol groups were quantified by referring to a calibration curve based on Cys standards (range: 127–1.275 µg/mL) prepared according to the same procedure. Thiol quantification was performed in triplicate and results are reported as means ± standard deviation.

### 4.5. Poly(Ether Urethane) Synthesis and Functionalization with Thiol Groups

The PEU used as synthetic counterpart of the bioartificial hydrogels designed in this work was synthesized according to an already optimized protocol [30,34]. Briefly, P407 (solubilized at 15% *w*/*v* in anhydrous DCE and equilibrated at 80 °C) was reacted with HDI at 1:2 molar ratio for 45 min in the presence of a catalytic amount of DBTDL (0.1% *w*/*w* with respect to P407). Then, the reaction mixture was quickly cooled to 60 °C and N-Boc added at 1:1 molar ratio with respect to P407 upon solubilization in anhydrous DCE at 5% *w*/*v*. After 2 h, the system was cooled to RT and the synthesis process was terminated through addition of MeOH. The synthesized PEU was collected by precipitation in excess petroleum ether and purified through solubilization in DCE followed by precipitation in a diethyl ether/MeOH mixture. The as-synthesized PEU will be referred to with the acronym DHP407, where D, H and P407 identify the constituent blocks, namely, N-Boc, HDI and P407, respectively.

In order to make DHP407 suitable for further functionalization through carbodiimide chemistry, the Boc-protected secondary amines present along its polymeric chains were made available through a Boc-deprotection reaction [30,51]. To this aim, DHP407 was subjected to an acidic treatment through solubilization at 4% *w*/*v* concentration in a mixture of chloroform (CF) and trifluoroacetic acid (TFA) at 90:10 *v*/*v*. In detail, the polymer was first solubilized in the CF aliquot for 2 h at RT and 250 rpm, then TFA was added and the reaction mixture stirred for an additional 60 min at 250 rpm. CF and TFA were evaporated under reduced pressure (Buchi Rotavapor Labortechnik AG, Flawil, Switzerland) and the collected polymer was purified by dialysis (regenerated cellulose membrane cut-off 10–12 kDa, Merck, Milan, Italy) for 48 h at 4 °C (3 complete refreshes per day) upon solubilization in demineralized water (10% *w*/*v* concentration). The Boc-deprotected DHP407 (referred to with the acronym D-DHP407) was finally collected through lyophilization (Martin Christ ALPHA 2-4 LSC, Osterode am Harz, Germany).

In order to expose thiol groups along the PEU chains, D-DHP407 was bulk-functionalized through carbodiimide chemistry by reacting the carboxylic acid group of TGA with the secondary amines exposed along D-DHP407 chains, leading to amide bond formation. The functionalization procedure was performed according to an already optimized protocol [34]. The carboxylic acid groups of TGA were first activated in an EDC/NHS aqueous solution (1:1 molar ratio between TGA-COOH and EDC/NHS) at pH 5 and 4 °C for 1 h under vigorous stirring. Then, a D-DHP407 aqueous solution (previously prepared in ddH_2_O and equilibrated at 4 °C) was added to the TGA solution at 1:20 -NH/-COOH molar ratio. The coupling reaction between amino and carboxylic acid groups was conducted at 4 °C and pH 5 for 6 h under stirring. Purification was carried out through dialysis (regenerated cellulose membrane cut-off 10–12 kDa, Merck, Milan, Italy) at 4 °C for 48 h using ddH_2_O with pH adjusted to 4 as dialysis medium. The purified thiolated PEU was finally collected through freeze-drying (Martin Christ ALPHA 2-4 LSC, Osterode am Harz, Germany) and referred to with the acronym S-DHP407.

### 4.6. Chemical Characterization of Thiol-Grafted PEU

The progressive development of a thiol-grafted PEU was monitored at all steps of the synthesis procedure. ATR-FTIR spectroscopy was performed on P407, DHP407, D-DHP407 and S-DHP407 according to the protocol adopted for functionalized HA characterization (Section 4.3.1).

The molecular weight distribution profile of the designed polymers was estimated through size exclusion chromatography (SEC) using an Agilent Technologies 1200 Series (CA, USA) equipped with HR1 and HR4 Waters Styragel columns and a concentration detector based on refractive index measurement. The adopted protocol has already been reported in our previous work [34]. The analyses were performed at each step of the synthesis procedure to assess not only the PEU successful synthesis but also the absence of degradation induced by the acidic conditions established during D-DHP407 and S-DHP407 preparation.

The exposure of secondary amines and thiol groups in D-DHP407 and S-DHP407, respectively, was assessed through colorimetric and spectroscopic analyses. In detail, D-DHP407 was characterized by the Orange II sodium salt assay and ^1^H NMR spectroscopy according to Laurano et al. [34]. Conversely, TGA grafting to S-DHP407 chains was assessed through NMR spectroscopy (both proton and carbon NMR analyses were conducted according to Laurano et al. [34]) and the amount of exposed thiol groups was quantified through the Ellman’s colorimetric assay as previously described [34].

### 4.7. Preliminary Evaluation of PEU-HA Miscibility and Injectability

To evaluate the miscibility of the synthetic and natural components, S-DHP407 and HA polymers were mixed at different weight ratios, i.e., 90/10, 80/20, 70/30, 60/40 and 50/50, by using a Luer lock double-syringe system. The overall polymeric concentration was kept constant at 15% *w*/*v* for all the considered formulations. Subsequently, samples were equilibrated at 4 °C and 37 °C and visually inspected at different time points (i.e., 1 h, 3 h, 24 h) to evaluate the occurrence of potential phase-separation phenomena. Afterward, bioartificial formulations were immersed in liquid nitrogen for 5 min and freeze-dried using a Martin Christ Alpha 2-4 LSC. Lastly, the network organization was morphologically investigated at the microscale through a scanning electron microscope (SEM) LEO 1450VP, at a beam voltage of 20.00 kV and 200X magnification.

Nanoscale characterization was also performed to evaluate potential effects induced by the presence of the natural component on the characteristic PEU capability to undergo a temperature-driven sol-to-gel transition [29,42] and to thoroughly study polymeric chain interactions. Specifically, dynamic light scattering (DLS) analysis was performed on S-DHP407, HA and S-DHP407/HA_50/50 *w*/*w* at 25 °C, 37 °C and 45 °C according to a previously reported method [29] using a Zetasizer Nano S90 (Malvern Instruments, Malvern, UK). Samples were prepared by dissolving the polymers (S-DHP407 or HA) at 0.5% *w*/*v* concentration in physiological saline solution (0.9% *w*/*v* NaCl) and then equilibrated at the test temperature overnight. S-DHP407/HA samples were prepared at 1% *w*/*v* in order to obtain systems with synthetic and natural polymer contents comparable to the corresponding controls. Analyses were performed on three different samples and the measured average hydrodynamic radii are reported as means ± standard deviation.

Lastly, the injectability of S-DHP407/HA formulations was also qualitatively assessed. Specifically, all the prepared systems were first equilibrated at 4 °C and RT and then injected through needles with different diameters (i.e., G18, G21 and G22) by three different potential users.

### 4.8. Bioartificial Hydrogel Preparation

Bioartificial hydrogels were prepared by first dissolving the synthetic and natural components in phosphate buffered saline (PBS, pH 7.4) solution at higher concentration, and then by mixing the obtained solutions through a Luer lock double-syringe system, thus leading to bioartificial formulations with an overall polymeric concentration of 15% *w*/*v*. In detail, to achieve a 1:1 functional group molar ratio and 50/50 PEU/HA weight ratio, the natural counterpart resulted in a mixture of functionalized and unfunctionalized HA. Only HA-DH and HA-SH functionalized according to the optimized conditions (as resulting from previous characterizations) were considered for bioartificial hydrogel preparation. To investigate the effects of an oxidizing agent on bioartificial hydrogel cross-linking kinetics, hydrogen peroxide (H_2_O_2_, 1:1 molar ratio with respect to HA functional groups) and horseradish peroxidase (HRP, 1:1 and 2:1 molar ratio with respect to HA functional groups) were also added to each formulation. Table 2 reports the content of each component to prepare 1 mL of each formulation and their corresponding acronyms.

### 4.9. Bioartificial Hydrogel Characterization

#### 4.9.1. Hydrogel Gelation at Physiological Temperature

Bioartificial hydrogel thermoresponsivity was investigated at the macroscale on all the designed formulations (15% *w*/*v*, 1 mL, *w*/and *w*/*o* H_2_O_2_ and HRP) prepared by mixing the two components at 50/50 *w*/*w* with a Luer lock double-syringe system as previously described. A DHP407/HA blend with similar composition (i.e., 50/50 *w*/*w*, 15% *w*/*v* overall concentration) was also prepared and tested as control. Then, formulations were kept in an incubator (Memmert IF75, Schwabach, Germany) at 37 °C and visually inspected at predefined time points (i.e., 10 min, 20 min, 30 min, 1 h, 2 h, 3 h, 4 h, 20 h, 22 h, 24 h and 48 h). The presence or absence of flow along the vial’s wall after 30 s of observation upon their capsizing were exploited to define the sol and gel state, respectively.

#### 4.9.2. Hydrogel Stability in Physiological-Mimicking Conditions

Hydrogel stability in a watery environment was tested in phosphate buffered saline (PBS, pH 7.4) at 37 °C by adapting an already developed method for purely thermosensitive PEU-based hydrogels [51]. The hydrogels (500 μL) were first prepared in glass vials with 10 mm internal diameter according to the protocol described in Section 4.8. After recording their initial weight (*W*_0_), the samples were equilibrated at 37 °C in a Memmert IF75 incubator for 45 min and then added with 500 μL of PBS previously heated at 37 °C. At predefined time intervals (30 min, 60 min, 90 min, 2 h, 3 h, 18 h, 20 h, 22 h and 24 h) the residual PBS was discarded, and the samples were weighed again to record the gel wet weight (*W_f_*). The percentage variation of wet weight was estimated at each time point using Equation (1).

(1) $$\mathrm{Wet}\;\mathrm{weight}\;\mathrm{change}\;\left(\%\right)=\frac{\left(W_{f}-W_{0}\right)}{W_{f}}\cdot 100$$

The analyses were conducted in triplicate and results are reported as means ± standard deviation. Purely thermoresponsive formulations unable to undergo in situ chemical cross-linking were also prepared using DHP407 and HA (overall polymeric concentration of 15% *w*/*v*, 50/50 *w*/*w*) and characterized according to the same protocol as control condition.

### 4.10. Statistical Analysis

Statistical analysis of the results was performed using GraphPad Prism version 5.03 for Windows (GraphPad Software, La Jolla, CA, USA; www.graphpad.com, accessed on 1 December 2022). Comparison among data was performed using *t*-tests with a 95% interval of confidence. For stability tests, differences among data were evaluated using two-way ANOVA coupled with Bonferroni’s multiple comparison test. Statistical differences were defined as reported by Boffito et al. [56].

## Figures and Tables

**Table 1:** Units of DH molecules per g of polymer measured from UV-vis and ^1^H NMR spectroscopic analyses performed on HA-DH_pHX samples. Percentages of DH molecules grafted to the HA backbone in the oxidized and unoxidized form.

	Units of DH/g of HA Measured from UV-vis	Units of DH/g of HA Measured from ^1^H NMR	Calculated Units of DH/g of HA in the Oxidized Form	% of Unoxidized DH	% of Oxidized DH
**HA-DH_pH5**	2.0 × 10^20^	1.9 × 10^20^	0.1 × 10^20^	95.0	5.0
**HA-DH_pH7**	2.5 × 10^20^	5.1 × 10^19^	1.9 × 10^20^	20.4	79.6
**HA-DH_pH9**	3.1 × 10^20^	6.1 × 10^19^	2.5 × 10^20^	19.7	80.3

**Table 2:** Nomenclature of the developed bioartificial blends based on S-DHP407 and HA-DH_pH5 (referred to as HA-DH) or HA-SH_pH5 (identified with the acronym HA-SH). Amounts of each component required for the preparation of 1 mL of bioartificial formulation. H_2_O_2_ was added to some formulations at 1:1 molar ratio with respect to HA thiol/catechol groups, while for HRP two different molar ratios with respect to HA functional groups (i.e., thiol or catechol groups) were tested (i.e., 1:1 and 2:1).

**S-DHP407/HA-DH Bioartificial Blends**					
**ACRONYM**	**S-DHP407 (mg)**	**HA-DH** **(mg)**	**HA** **(mg)**	**H_2_O_2_** **(mol_H2O2_:mol_cathecol_)**	**HRP** **(U/mL)**
S-DHP407/HA-DH	75	15	60	-	-
S-DHP407/HA-DH_HRP_1	75	15	60	1:1	4 (mol_HRP_:mol_cathecol_ = 1:1)
S-DHP407/HA-DH_HRP_2	75	15	60	1:1	8 (mol_HRP_:mol_cathecol_ = 2:1)
**S-DHP407/HA-SH Bioartificial Blends**					
**ACRONYM**	**S-DHP407** **(mg)**	**HA-SH** **(mg)**	**HA** **(mg)**	**H_2_O_2_** **(mol_H2O2_:mol_thiol_)**	**HRP** **(U/mL)**
S-DHP407/HA-SH	75	15	60	-	-
S-DHP407/HA-SH_HRP_1	75	15	60	1:1	4 (mol_HRP_:mol_thiol_ = 1:1)
S-DHP407/HA-SH_HRP_2	75	15	60	1:1	8 (mol_HRP_:mol_thiol_ = 2:1)

## Data Availability

Not applicable.

## Supplementary Materials

The following supporting information can be downloaded at https://www.mdpi.com/article/10.3390/gels9010059/s1. Video S1: Bioartificial hydrogel preparation through a double-syringe system. Figure S1: (a) Ellman’s colorimetric assay performed on HA (left) and HA-SH_pH5 (right): the strong yellowish color of HA-SH_pH5 compared to HA (control) proves the successful exposure of sulfhydryl groups along Cys-functionalized HA; (b) Number of exposed –SH groups along HA-SH_pHX samples as quantified through the Ellman’s colorimetric assay. Results for HA-SH_pH4, HA-SH_pH5 and HA-SH_pH7 are reported in light blue, blue and dark blue, respectively. Figure S2: SEM images of samples based on virgin polymers (i.e., S-DHP407 and HA) and on their 50/50 *w*/*w* blend. Scale bar: 100 µm. Figure S3: Representative photo of the Luer lock double-syringe system used for bioartificial hydrogel preparation. To ensure complete mixing, polymeric formulations were pipetted up and down 5 times.

## References

[1] González-Díaz E.C., Varghese S. (2016). Hydrogels as extracellular matrix analogs. Gels.

[2] Ahmed E.M. (2015). Hydrogel: Preparation, characterization, and applications: A review. J. Adv. Res..

[3] Shi J., Votruba A.R., Farokhzad O.C., Larger R. (2010). Nanotechnology in drug delivery and tissue engineering: From discovery to applications. Nano Lett..

[4] Jacob S., Nair A.B., Shah J., Sreeharsha N., Gupta S., Shinu P. (2021). Emerging Role of Hydrogels in Drug Delivery Systems, Tissue Engineering and Wound Management. Pharmaceutics.

[5] Sun Y., Nan D., Jin H., Qu X. (2020). Recent advances of injectable hydrogels for drug delivery and tissue engineering applications. Polym. Test..

[6] Bustamante-Torres M., Romero-Fierro D., Arcentales-Vera B., Palomino K., Magaña H., Bucio E. (2021). Hydrogels classification according to the physical or chemical interactions and as stimuli-sensitive materials. Gels.

[7] Turturro M.V., Sokic S., Larson J.C., Papavasiliou G. (2013). Effective tuning of ligand incorporation and mechanical properties in visible light photopolymerized poly(ethylene glycol) diacrylate hydrogels dictates cell adhesion and proliferation. Biomed. Mater..

[8] Wu S., Hua M., Alsaid Y., Du Y., Ma Y., Zhao Y., Lo C.Y., Wang C., Wu D., Yao B. (2021). Poly(vinyl alcohol) Hydrogels with Broad-Range Tunable Mechanical Properties via the Hofmeister Effect. Adv. Mater..

[9] Liu B., Hou Z., Bao Y., Hua L., Wang X., Li Y., Zhou L., Lv Z. (2022). Tuning Mechanical Properties of Polymeric Hydrogels Using Orthogonally Functionalized Crosslinkers. Adv. Polym. Technol..

[10] Liu C., Zhang Q., Zhu S., Liu H., Chen J. (2019). Preparation and applications of peptide-based injectable hydrogels. RSC Adv..

[11] Pramanik B. (2022). Short peptide-based smart thixotropic hydrogels. Gels.

[12] Park H., Choi B., Hu J., Lee M. (2013). Injectable chitosan hyaluronic acid hydrogels for cartilage tissue engineering. Acta Biomater..

[13] Teixeira Cerqueira M., Pereira da Silva L., Santos T.C., Pirraco R.P., Correlo V.M., Reis R.L., Pinto Marques A. (2014). Gum-Hyaluronic Acid Spongy-like Hydrogels and Cells from Adipose Tissue Synergize Promoting Neoskin Vascularization. ACS Appl. Mater. Interfaces.

[14] Portalska K.J., Teixeira L.M., Leijten J.C.H., Jin R., Blitterswijk C., Boer J., Karperien M. (2014). Boosting Angiogenesis and Functional Vascularization in Injectable Dextran–Hyaluronic Acid Hydrogels by Endothelial-Like Mesenchymal Stromal Cells. Tissue Eng. Part A.

[15] Pourjavadi A., Tavakolizadeh M., Hosseini S.H., Rabiee N., Bagherzadeh M. (2020). Highly stretchable, self-adhesive, and self-healable double network hydrogel based on alginate/polyacrylamide with tunable mechanical properties. J. Polym. Sci..

[16] Lin T., Bai Q., Peng J., Xu L., Li J., Zhai M. (2018). One-step radiation synthesis of agarose/polyacrylamide double-network hydrogel with extremely excellent mechanical properties. Carbohydr. Polym..

[17] Thankam F.J., Muthu J., Sankar V., Gopal R.K. (2013). Growth and survival of cells in biosynthetic poly vinyl alcohol–alginate IPN hydrogels for cardiac applications. Colloids Surf. B.

[18] Mellati A., Akhtari J. (2018). Injectable hydrogels: A review of injectability mechanisms and biomedical applications. Res. Mol. Med..

[19] Bertsch P., Diba M., Mooney D.J., Leeuwenburgh S.C.G. (2022). Self-healing injectable hydrogels for tissue regeneration. Chem. Rev..

[20] Alonso J.M., Andrade Del Olmo J., Perez Gonzalez R., Saez-Martinez V. (2021). Injectable hydrogels: From laboratory to industrialization. Polymers.

[21] Ren P., Wei D., Ge X., Wang F., Liang M., Dai J., Xu L., Zhang T. (2021). Injectable supramolecular hydrogels based on host–guest interactions with cell encapsulation capabilities. Colloids Surf. A.

[22] Deng Z., He Y., Wang Y.J., Zhao Y., Chen L. (2020). Chondroitin sulfate hydrogels based on electrostatic interactions with enhanced adhesive properties: Exploring the bulk and interfacial contributions. Soft Matter.

[23] Shi J., Guo-bao W., Chen H., Zhong W., Qiu X., Xing M.M. (2014). Schiff based injectable hydrogel for in situ pH-triggered delivery of doxorubicin for breast tumor treatment. Polym. Chem..

[24] Fan H., Wang J., Jin Z. (2018). Tough, Swelling-Resistant, Self-Healing, and Adhesive Dual-Cross-Linked Hydrogels Based on Polymer–Tannic Acid Multiple Hydrogen Bonds. Macromolecules.

[25] Chen M., Ren X., Dong L., Li X., Cheng H. (2021). Preparation of dynamic covalently crosslinking keratin hydrogels based on thiol/disulfide bonds exchange strategy. Int. J. Biol. Macromol..

[26] Casuso P., Odriozola I., Vicente A.P., Loinaz I., Cabañero G., Grande H.J., Damien D. (2015). Injectable and Self-Healing Dynamic Hydrogels Based on Metal(I)-Thiolate/Disulfide Exchange as Biomaterials with Tunable Mechanical Properties. Biomacromolecules.

[27] Wei H., Li S., Liu Z., Chen H., Liu Y., Li W., Wang G. (2022). Preparation and characterization of starch-cellulose interpenetrating network hydrogels based on sequential Diels-Alder click reaction and photopolymerization. Int. J. Biol. Macromol..

[28] Wei Z., Yang J.H., Du X.J., Xu F., Zrinyi M., Osada Y., Li F., Chen Y.M. (2013). Dextran-based self-healing hydrogels formed by reversible diels-alder reaction under physiological conditions. Macromol. Rapid Commun..

[29] Laurano R., Abrami M., Grassi M., Ciardelli G., Boffito M., Chiono V. (2020). Using Poloxamer^®^ 407 as Building Block of Amphiphilic Poly(ether urethane)s: Effect of its Molecular Weight Distribution on Thermo-Sensitive Hydrogel Performances in the Perspective of Their Biomedical Application. Front. Mater..

[30] Laurano R., Chiono V., Ceresa C., Fracchia L., Zoso A., Ciardelli G., Boffito M. (2021). Custom-design of intrinsically antimicrobial polyurethane hydrogels as multifunctional injectable delivery systems for mini-invasive wound treatment. Eng. Regen..

[31] Kaya G., Tran C., Sorg O., Hotz R., Grand D., Carraux P., Didierjean L., Stamenkovic I., Saurat J.H. (2006). Hyaluronate fragments reverse skin atrophy by a CD44-dependent mechanism. PLoS Med..

[32] Slevin M., Kumar S., Gaffney J. (2002). Angiogenic oligosaccharides of hyaluronan induce multiple signaling pathways affecting vascular endothelial cell mitogenic and wound healing responses. J. Biol. Chem..

[33] Papakonstantinou E., Roth M., Karakiulakis G. (2012). Hyaluronic acid: A key molecule in skin aging. Dermato-Endocrinology.

[34] Laurano R., Cassino C., Ciardelli G., Chiono V., Boffito M. (2020). Polyurethane-based thiomers: A new multifunctional copolymer platform for biomedical applications. React. Funct. Polym..

[35] Lih E., Choi S.G., Ahn D.J., Joung Y.K., Han D.K. (2016). Optimal conjugation of catechol group onto hyaluronic acid in coronary stent substrate coating for the prevention of restenosis. J. Tissue Eng..

[36] Nguyen L.T.B., Hsu C.C., Ye H., Cui Z. (2020). Development of an in situ injectable hydrogel containing hyaluronic acid for neural regeneration. Biomed. Mater..

[37] Barreto W.J., Ponzoni S., Sassi P. (1998). A Raman and UV-Vis study of catecholamines oxidized with Mn(III). Spectrochim. Acta Part A Mol. Biomol. Spectrosc..

[38] Melnik T., Ben Ameur S., Kanfar N., Vinet L., Delie F., Jordan O. (2022). Bioadhesive hyaluronic acid/dopamine hydrogels for vascular applications prepared by initiator-free crosslinking. Int. J. Mol. Sci..

[39] Yu J., Wei W., Menyo M.S., Masic A., Waite J.H., Israelachvili J.N. (2013). Adhesion of mussel foot protein-3 to TiO2 surfaces: The Effect of pH. Biomacromolecules.

[40] Laffleur F., Psenner J., Suchaoin W. (2015). Permeation enhancement via thiolation: In vitro and ex vivo evaluation of hyaluronic acid-cysteine ethyl ester. J. Pharm. Sci..

[41] Anitha A., Deepa N., Chennazhi K.P., Nair S.V., Tamura H., Jayakumar R. (2011). Development of mucoadhesive thiolated chitosan nanoparticles for biomedical applications. Carbohydr. Polym..

[42] Winther J.R., Thorpe C. (2014). Quantification of thiols and disulfides. Biochim. Biophys. Acta..

[43] Chan K.Y., Wasserman B.P. (1993). Direct colorimetric assay of free thiol groups and disulfide bonds in suspensions of solubilized and particulate cereal proteins. Cereal. Chem..

[44] Laurano R., Boffito M. (2020). Thermosensitive Micellar Hydrogels as Vehicles to Deliver Drugs With Different Wettability. Front. Bioeng. Biotechnol..

[45] Xu F., Nacker J.C., Crone W.C., Masters K.S. (2008). The haemocompatibility of polyurethane-hyaluronic acid copolymers. Biomaterials.

[46] Sakulwech S., Lourith N., Ruktanonchai U., Kanlayavattanakul M. (2018). Preparation and characterization of nanoparticles from quaternized cyclodextrin-grafted chitosan associated with hyaluronic acid for cosmetics. Asian J. Pharm. Sci..

[47] Singh S., Topuz F., Hahn K., Albrecht K., Groll J. (2013). Embedding of Active Proteins and Living Cells in Redox-Sensitive Hydrogels and Nanogels through Enzymatic Cross-Linking. Angew. Chem. Int. Ed..

[48] Lee F., Hyun Bae K., Kurisawa M. (2016). Injectable hydrogel systems crosslinked by horseradish peroxidase. Biomed. Mater..

[49] Bae J.W., Choi J.H., Lee Y., Park K.D. (2015). Horseradish peroxidase-catalysed in situ-forming hydrogels for tissue-engineering applications. J. Tissue Eng. Regen. Med..

[50] Park H.J., Jin Y., Shin J., Yang K., Lee C., Yang H.S., Cho S.W. (2016). Catechol-functionalized hyaluronic acid hydrogels enhance angiogenesis and osteogenesis of human adipose-derived stem cells in critical tissue defects. Biomacromolecules.

[51] Šoltés L., Kogan G., Stankovská M., Mendichi R., Schiller J., Gemeiner P. (2007). Degradation of high-molar-mass hyaluronan and characterization of fragments. Biomacromolecules.

[52] Meijs G.F., McCarthy S.J., Rizzardo E., Chen Y.C., Chatelier R.C., Brandwood A., Schindhelm K. (1993). Degradation of medical-grade polyurethane elastomers: The effect of hydrogen peroxide in vitro. J. Biomed. Mater. Res..

[53] Boffito M., Torchio A., Tonda-Turo C., Laurano R., Gisbert-Garzarán M., Berkmann J.C., Cassino C., Manzano M., Duda G.N., Vallet-Regí M. (2020). Hybrid injectable sol-gel systems based on thermo-sensitive polyurethane hydrogels carrying pH-sensitive mesoporous silica nanoparticles for the controlled and triggered release of therapeutic agents. Front. Bioeng. Biotechnol..

[54] Laurano R., Boffito M., Abrami M., Grassi M., Zoso A., Chiono V., Ciardelli G. (2021). Dual stimuli-responsive polyurethane-based hydrogels as smart drug delivery carriers for the advanced treatment of chronic skin wounds. Bioact. Mater..

[55] Lin C.Y., Liu J.C. (2020). Comparison between Catechol- and Thiol-Based Adhesion Using Elastin-like Polypeptides. ACS Appl. Bio Mater..

[56] Boffito M., Gioffredi E., Chiono V., Calzone S., Ranzato E., Martinotti S., Ciardelli G. (2016). Novel polyurethane-based thermosensitive hydrogels as drug release and tissue engineering platforms: Design and in vivo characterization. Polym. Int..

