# Supplementary material for: Design of Injectable Bioartificial Hydrogels by Green Chemistry for Mini-Invasive Applications in the Biomedical or Aesthetic Medicine Fields
Source: Gels. 2023 Jan 11;9(1):59. doi: 10.3390/gels9010059 (PMC9858130; doi:10.3390/gels9010059)
Supplement: Supplementary file 1 [file gels-09-00059-s001.zip › Supplementary material/supplementary material.pdf]

Design of Injectable Bioartificial Hydrogels by Green Chemistry for Mini-Invasive Applications in the Biomedical or Aesthetic Medicine Fields

Rossella Laurano [1,§,*], Monica Boffito [1,2,§], Claudio Cassino [3], Francesco Liberti[1], Gianluca Ciardelli [1,2,#] and Valeria Chiono [1,2,#]

[1] Department of Mechanical and Aerospace Engineering, Politecnico di Torino, 10129, Torino, Italy
[2] Institute for Chemical-Physical Processes, National Research Council (CNR-IPCF), 56124, Pisa, Italy
[3] Department of Science and Technological Innovation, Università del Piemonte Orientale, 15121, Alessandria, Italy
* Correspondence: rossella.laurano@polito.it;
§ These authors equally contributed to this work
# These authors share the last co-authorship

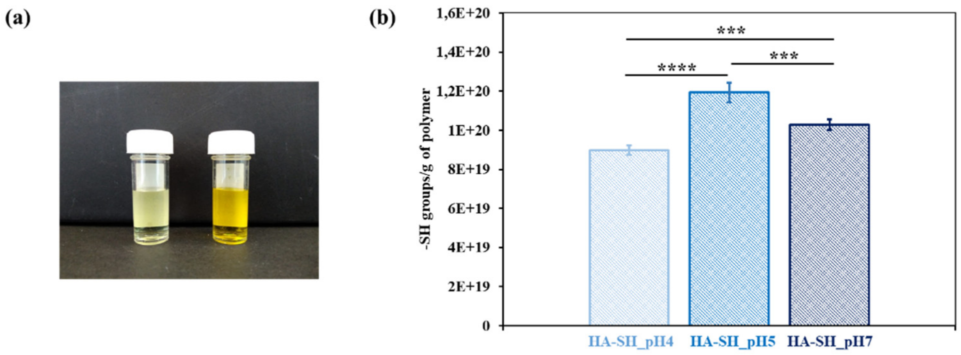

Figure S1. (a) Ellman's colorimetric assay performed on HA (left) and HA-SH_pH5 (right): the strong yellowish color of HA-SH_pH5 compared to HA (control) proves the successful exposure of free sulfhydryl groups along Cys-functionalized HA; (b) Number of exposed –SH groups along HA-SH_pHX samples as quantified through the Ellman's colorimetric assay. Results for HA-SH_pH4, HA-SH_pH5 and HA-SH_pH7 are reported in light blue, blue and dark blue, respectively.

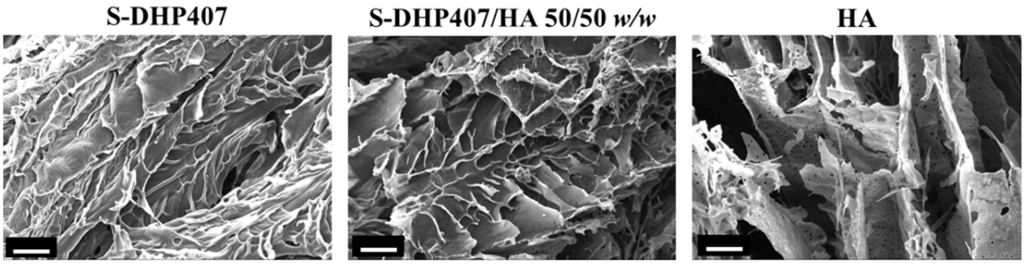

Figure S2. SEM images of samples based on virgin polymers (i.e., S-DHP407 and HA) and on their 50/50 *w/w* blend. Scale bar: 100 μm.

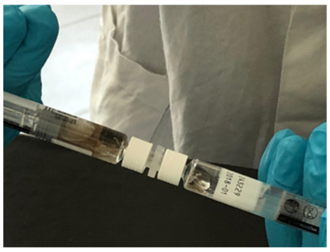

Figure S3. Representative photo of the Luer-lock double syringe system used for bioartificial hydrogel preparation. To ensure complete mixing, polymeric formulations were pipetted up and down 5 times.
